# Systematic evaluation of incomplete penetrance in 4,325 rare disease families reveals divergent transcriptional networks in *CHD8* pathology

**DOI:** 10.64898/2026.10.05.26364218

**Published:** 2026-10-07

**Authors:** Mugdha Singh, Sarah L. Stenton, Emily Groopman, Moriel Singer-Berk, Jialan Ma, Sanaa Choufani, Emily O’Heir, Stephanie DiTroia, Ikeoluwa Osei-Owusu, Lynn Pais, Gabrielle Lemire, Melanie O’Leary, Christina Austin-Tse, Casie A. Genetti, Katherine N. Anderson, Monica H. Wojcik, Alan H. Beggs, Jennifer E. Neil, Abbe Lai, Christopher A. Walsh, Julie A. Jurgens, Brenda J. Barry, Elizabeth C. Engle, Sandra Donkervoort, Carsten G. Bönnemann, Emanuela Argilli, Elliott H. Sherr, Patricia Kipkemoi, Zandre Bruwer, Kirsten A. Donald, Volker Straub, Ana Topf, Susan M. White, Tiong Yang Tan, Lyndon Gallacher, Peers Arts, Matilda R. Jackson, Thuong T. Ha, Christopher P. Barnett, Alicia B. Byrne, Hamish S. Scott, Vijay S. Ganesh, Heidi L. Rehm, Rosanna Weksberg, Anne O’Donnell-Luria, Sanna Gudmundsson

## Abstract

**INTRODUCTION:**

Despite major advances in genomic sequencing and analytical pipelines for rare disorders, more than 50% of affected individuals remain without a molecular diagnosis. One challenge is the interpretation of inherited variants exhibiting incomplete penetrance (IP).

**MATERIALS AND METHODS:**

We developed a pipeline to systematically investigate inherited variants as the cause of rare disease in 4,325 families, consisting of affected probands and unaffected parents, from the Broad Center for Mendelian Genomics, part of the GREGoR Consortium. As a proof-of-concept of further omics follow-up, epigenomic and transcriptomic analyses were performed in one family harboring an inherited disease-causing frameshift variant in *CHD8* .

**RESULTS:**

We identified 81 families where the affected proband harbored a putative disease-causing variant inherited from a reportedly unaffected parent. Clinical re-evaluation identified 30 families in which an inherited variant was considered diagnostic, including six previously missed diagnoses. In this cohort, inherited IP variants account for 1.6% of the diagnosed cases, with an overall diagnostic yield of 0.4% in the cohort. Methylation analysis of the *CHD8* family confirmed a positive *CHD8* clinical episignature in the male proband, but also in the reportedly unaffected mother. Transcriptomic analysis showed that despite similar *CHD8* haploinsufficiency, the proband and mother displayed divergent downstream transcriptional effects.

**DISCUSSION:**

This study highlights the importance of considering parentally inherited IP variants in rare disease, with at least 1 in 60 diagnostic variants inherited from a reportedly unaffected parent, and demonstrates the potential of integrating omics evaluation to improve variant interpretation.

## Full Text Availability

The license terms selected by the author(s) for this preprint version do not permit archiving in PMC. The full text is available from the preprint server.

